# Unexpected voriconazole toxicity due to nirmatrelvir/ritonavir: a case report on drug-drug interaction and the role of therapeutic drug monitoring

**DOI:** 10.3389/fphar.2025.1616061

**Published:** 2025-06-12

**Authors:** J. López-Hernández, A. B. Guisado-Gil, M. Mejías-Trueba, L. Herrera-Hidalgo, F. J. Reina-Martínez, M. V. Gil-Navarro

**Affiliations:** ^1^ Department of Pharmacy, Virgen del Rocío University Hospital, Seville, Spain; ^2^ Department of Infectious Diseases, Microbiology and Parasitology, Virgen del Rocío University Hospital, Seville, Spain; ^3^ Institute of Biomedicine of Seville, Virgen del Rocío University Hospital/CSIC/University of Seville, Seville, Spain; ^4^ Centro de Investigación Biomédica en Red de Enfermedades Infecciosas (CIBERINFEC), Madrid, Spain; ^5^ Intensive Care Medicine Department, Virgen del Rocío University Hospital, Seville, Spain

**Keywords:** voriconazole, aspergillosis, COVID-19, drug-drug interactions, therapeutic drug monitoring

## Abstract

Voriconazole is a triazole antifungal used for invasive fungal infections, particularly invasive aspergillosis. Its metabolism is primarily mediated by CYP2C19, with CYP3A4 and CYP2C9 also involved. Nirmatrelvir/ritonavir, an oral antiviral for COVID-19, inhibits CYP isoforms potentially altering the metabolism of co-administered drugs. We report a case of an immunosuppressed patient with SARS-CoV-2 pneumonia and invasive aspergillosis treated with voriconazole and nirmatrelvir/ritonavir. Unexpectedly, voriconazole plasma concentrations increased significantly (7.78 mg/L) instead of the anticipated decrease, leading to temporary discontinuation. Therapeutic drug monitoring (TDM) guided dose adjustments until optimal levels (2 mg/L) were achieved. After 13 days, the patient recovered from COVID-19, with clinical improvement of aspergillosis. This case highlights the importance of pharmacokinetic monitoring and drug-drug interaction assessment in critically ill patients.

## Introduction

Voriconazole is a triazole antifungal agent indicated for the treatment of invasive fungal infections, particularly invasive aspergillosis. It undergoes a complex metabolism mediated by different isoforms of cytochrome P450. The main enzyme responsible for its metabolism is CYP2C19, although CYP3A4 and CYP2C9 are also involved (Barbarino et al., 2017).

Nirmatrelvir/ritonavir is an oral antiviral agent indicated for the treatment of coronavirus disease 2019 (COVID-19) in adults who do not require supplemental oxygen but are at high risk of developing severe illness. It consists of the combination of nirmatrelvir, a SARS-CoV-2 main protease (Mpro) inhibitor, and ritonavir, which is used for its action as a CYP3A4 inhibitor, which reduces the metabolism of nirmatrelvir and increases its plasma levels (ema.europa, 2024).

We present a case report of a patient who received concomitant voriconazole and nirmatrelvir/ritonavir during hospitalization. Nirmatrelvir/ritonavir caused a significant increase in voriconazole plasma concentrations, and the patient developed signs and symptoms of voriconazole toxicity. The case was reported to the Spanish Human Medicines Pharmacovigilance System.

## Case

We present the case of a 71-year-old man, weighing 74 kg and measuring 170 cm, who was receiving rituximab (last dose 1 month prior to admission) and prednisone (30 mg/day) for optic neuromyelitis diagnosed 2 months prior to admission, without other relevant comorbidities. The patient was not an organ transplant recipient. He was hospitalized some weeks earlier with bilateral pneumonia due to SARS-CoV-2 infection, despite having received COVID-19 vaccination, requiring high-flow oxygen (HFO) therapy to maintain oxygen saturations of 92%–93%. The patient received a 5 days course of nirmatrelvir/ritonavir. After improvement he was discharged with oxygen therapy and home follow-up.

Six days after discharge, he returned to the Emergency Department with dyspnea, fever of 38°C, and asthenia. While in the Observation Unit, he developed acute hypoxemic respiratory failure. The COVID-19 PCR (Polymerase Chain Reaction) test was again positive (cycle threshold (CT) 18), and laboratory tests revealed neutrophilia and elevated C-reactive protein (200 mg/L). Chest X-ray showed worsening compared to previous imaging. His oxygen saturation dropped to 88% despite continued HFO, and he was admitted to the Intensive Care Unit (ICU) for stabilization and intubation. A fibrobronchoscopy with microbiological examination of bronchoaspirate was performed, and treatment was started with piperacillin/tazobactam, and dexamethasone. Given the severity of the SARS-CoV-2 infection and the patient’s immunosuppressed status, an off-label retreatment with nirmatrelvir/ritonavir for 10 days was also initiated (the patient had previously received a 5 days course of nirmatrelvir/ritonavir during his first admission) in the context of an intrahospital protocol approved by the Pharmacy Commission that contemplates the use of nirmatrelvir/ritonavir in patients with pneumonia.

The next day, invasive pulmonary aspergillosis was confirmed as a superinfection (positive galactomannan test), prompting the initiation of intravenous voriconazole at 6 mg/kg (444 mg) every 12 h for the first two doses, followed by 4 mg/kg (296 mg) every 12 h as maintenance therapy. Vancomycin was also added for suspected Gram-positive bacterial superinfection. Within 24 h, acute-phase reactants decreased, and the patient became afebrile.

**FIGURE 1:**
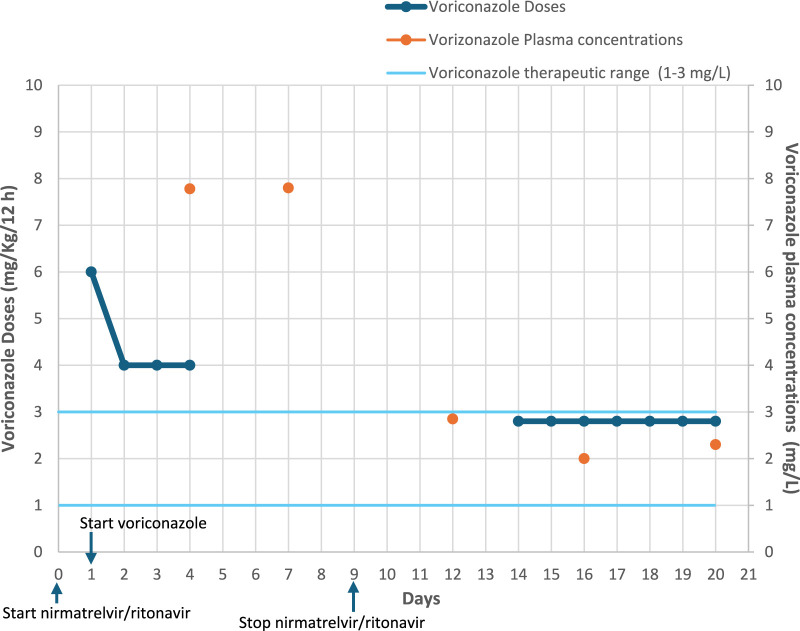
Voriconazole doses and plasma concentrations.

After four days of voriconazole therapy, plasma concentration (Cp) monitoring revealed supratherapeutic levels (7.78 mg/L). Concerned about this finding, the physician contacted the Pharmacy Service to request therapeutic drug monitoring (TDM) for individualized dosing to achieve a targetCp of 1–3 mg/L (Figure 1). A bayesian estimation of individual pharmacokinetic parameters was conducted using Rx Studio software. Pharmacists reviewed the patient´s treatment regimen and assessed potential drug-drug interactions. A potential interaction between nirmatrelvir/ritonavir and voriconazole was detected, described in several databases (Liverpool COVID-19, Micromedex and Lexicomp). Interestingly, the expected interaction between nirmatrelvir/ritonavir and voriconazole is typically a decrease in voriconazole plasma levels, due to ritonavir-induced CYP2C19 activation, the main CYP isoform responsible for the metabolism of the antifungal drug. However, the opposite effect was observed in this patient, with unexpectedly elevated voriconazole levels.

After TDM analysis, it was estimated that therapeutic Cp levels would be reached after 72 h without voriconazole. Based on this, treatment was temporarily stopped, and a new Cp measurement was scheduled. However, after 72 h, voriconazole Cp remained elevated at 7.80 mg/L, leading to the decision to discontinue voriconazole until completion of nirmatrelvir/ritonavir treatment and confirmation that plasma levels had returned to a therapeutic range.

Two days after the end of antiviral treatment, voriconazole Cp was measured and found to be in the therapeutic range (2.85 mg/L). However, due to hypertransaminasemia, it was decided to delay reintroduction of voriconazole until 4 days after the end of nirmatrelvir/ritonavir. At that time, voriconazole was restarted at 200 mg every 12 h. Two days after restarting voriconazole, a repeat Cp measurement was performed and the result was 2 mg/L. As this was within the target range, it was decided to maintain the 200 mg every 12 h regimen with less frequent monitoring. A follow-up Cp measurement 4 days later, confirmed therapeutic levels (2.3 mg/L).

Thirteen days after completing nirmatrelvir/ritonavir therapy, the patient was symptom-free from COVID-19 infection, and viremia was negative 40 days after symptom onset. In addition, there were no residual radiologic infiltrates or signs of respiratory failure. Regarding invasive aspergillosis, the patient showed favorable clinical progression and remained on voriconazole 200 mg/12 h orally until the completion of treatment 1 month later.

Finally, the patient was transferred to the general ward and, upon discharge, continued follow-up with the Infectious Diseases Service through outpatient consultations.

## Discussion

Nirmatrelvir/ritonavir was approved in Europe by the European Medicines Agency in January 2022 for the treatment of COVID-19 in adults who do not require supplemental oxygen but are at increased risk of developing severe disease (Agency EM, 2022). It is currently approved in more than 70 countries worldwide. The standard regimen consists of 300 mg of nirmatrelvir plus 100 mg of ritonavir administered twice daily for 5 days, with dose adjustments based on renal function (Butt et al., 2024). In certain cases, particularly in patients with severe SARS-Cov-2 infection or pneumonia, off-label use includes extending the duration of treatment to 10 days (Snell et al., 2024).

Voriconazole has a high potential for drug-drug interactions, especially with CYP3A4-metabolized drugs. Consequently, it is contraindicated with drugs that are highly dependent on CYP3A4 for elimination, especially those whose elevated Cp is associated with life-threatening adverse reactions (ema.europa, 2024). Coadministration of voriconazole or another antifungal drug with antiviral drugs used for COVID-19 should be avoided and only be used when the benefit-risk balance is positive (Spanakis et al., 2021), especially in elderly polymedicated patients, since these are the patients who most frequently experience drug interactions and adverse drug reactions (Crescioli et al., 2021). In addition, voriconazole is an antifungal agent with a narrow therapeutic range and high intra- and interindividual variability influenced by factors such as liver function, age, weight, CYP2C19 polymorphisms, inflammation, and drug interactions. Due to these variables, TDM of voriconazole is essential to optimize efficacy while minimizing adverse effects such as neurotoxicity and hepatotoxicity (Veringa et al., 2023).

The main drug interaction databases (Liverpool COVID-19, Micromedex, and Lexicomp) indicate that co-administration of nirmatrelvir/ritonavir and voriconazole leads to a decrease in voriconazole Cp. This is attributed to the inducing effect of ritonavir on CYP2C19, the primary enzyme responsible for voriconazole metabolism. While ritonavir is a potent CYP3A4 inhibitor, at high doses, it also induces CYP2C19 and CYP2C9, which further contribute to voriconazole metabolism. This dual effect (enzyme inhibition and induction) ultimately results in a potential reduction in voriconazole Cp when ritonavir is used at high doses (Loos et al., 2022). A 2007 study by Liu et al. concluded that voriconazole exposure, measured as the area under the curve (AUC), was reduced by 82% when co-administered with ritonavir 400 mg every 12 h for 9 days. Conversely, a lower dose of ritonavir (100 mg every 12 h) resulted in a moderate AUC reduction of approximately 39% (Liu et al., 2007).

However, in our case, voriconazole Cp increased and reached supratherapeutic levels when co-administered with nirmatrelvir/ritonavir, ultimately leading to treatment discontinuation. A similar case published in 2023 described a significant increase in voriconazole Cp when both drugs were co-administered. The authors suggested that, due to the short duration (5 days) of nirmatrelvir/ritonavir treatment, its CYP3A4 inhibitory effects prevailed over CYP2C19 induction, leading to elevated voriconazole levels (Bayo et al., 2023). In our case, treatment lasted 10 days instead of 5, yet the patient still experienced supratherapeutic voriconazole levels and signs of toxicity (hypertransaminasemia). This paradoxical increase may be explained by the rapid onset of CYP3A4 inhibition by ritonavir, which occurs in hours to days dominating during the early phase of treatment and leading to an increase in voriconazole Cp (Reitman et al., 2011).

However, CYP2C19 induction is a slower process that typically takes several days to weeks to reach maximum effect, depending on factors such as protein synthesis rates and the half-life of the inducer (Reitman et al., 2011). It is possible that even with 10 days of nirmatrelvir/ritonavir treatment, CYP2C19 induction takes longer to develop while CYP3A4 inhibition occurs more rapidly. This may explain the unexpected increase in voriconazole Cp observed in our patient.

An additional explanation for the variation in plasma levels could be found in the field of pharmacogenetics. Knowing the patient´s genotype could have helped explain the observed pharmacokinetics and provided valuable insight into their risk of elevated plasma concentrations, however, at the moment, pharmacogenetics analysis was not available in our centre.

In clinical practice, the study of the patient´s genotype may support more accurate initial dosing, depending on whether the patient is an ultrarapid metabolizer and therefore presents reduced voriconazole concentrations with usual doses; or a slow metabolizer, who presents high voriconazole concentrations than expected, and a greater risk of adverse events (Moriyama et al., 2017). Despite our patient was not a transplant recipient, due to high medication burden and frequent use of immunosuppressive agents and antifungal prophylaxis, these findings may be relevant and potentially generalizable to transplant populations. In the context of complex drug-drug and drug-gene interactions (Gasche et al., 2004), and high interindividual variability, genotyping may enhance personalized approaches and improve patient safety.

In conclusion, monitoring voriconazole Cp is crucial when drug interactions are suspected, as they can significantly impact efficacy and safety. While some interactions can be predicted based on pharmacokinetic or pharmacodynamic mechanisms, others remain unpredictable due to variables such as genetics, age, comorbidities, polypharmacy, and treatment duration. TDM enables real-time dose adjustments, ensuring a personalized treatment approach, optimizing clinical outcomes and enhancing patient safety.

## Data Availability

The original contributions presented in the study are included in the article/supplementary material, further inquiries can be directed to the corresponding author.
